# Serological Profile of Hepatitis B, C, and D and Factors Associated With Hepatitis B Exposure and Vaccination Among Hemodialysis Patients in Northeastern Brazil

**DOI:** 10.1155/ijne/4932261

**Published:** 2026-08-28

**Authors:** Lucas Lima da Silva, Jurema Correia Mota, Alanna Calheiro dos Santos, Vanessa Duarte da Costa, Giselle Prado do Nascimento, Julia Trece Marques, Juliana Custodio Miguel, Alexandre Braga Liborio, Danielle Malta Lima, Jeová Keny Baima Colares, Vanessa Salete de Paula, Livia Melo Villar

**Affiliations:** ^1^ Viral Hepatitis Laboratory, Oswaldo Cruz Institute, Fiocruz, Rio de Janeiro, Brazil, fiocruz.br; ^2^ Graduate Program in Medical Sciences, Universidade de Fortaleza (UNIFOR), Fortaleza, Ceará, Brazil, unifor.br; ^3^ Molecular Virology and Parasitology Laboratory, Oswaldo Cruz Institute, Oswaldo Cruz Foundation, Rio de Janeiro, Brazil, fiocruz.br

**Keywords:** chronic kidney disease, hemodialysis, hepatitis B, hepatitis C, liver

## Abstract

**Introduction:**

Hemodialysis patients are at increased risk for viral hepatitis due to treatment‐related exposures and transmission routes, potentially worsening liver and kidney disease. However, updated regional data in Brazil are scarce. This study evaluated the seroprevalence of hepatitis B, C, and D viruses (HBV, HCV, and HDV) and analyzed risk factors associated with HBV infection among patients undergoing hemodialysis in Northeast Brazil.

**Methods:**

This study corresponds to a cross‐sectional evaluation of 293 patients from two hemodialysis clinics. Serological markers (HBsAg, anti‐HDV, anti‐HBc, anti‐HBs, and anti‐HCV) were analyzed by chemiluminescence, and the quantification of viral load was measured by real‐time PCR. Sociodemographic and risk‐related characteristics were collected via questionnaire. Statistical analyses included chi‐square and Fisher tests (*p* < 0.05).

**Results:**

The prevalence of hepatitis B (HBsAg+) and hepatitis C (anti‐HCV+) was 2.4% and 4.1%, respectively. Regarding hepatitis B serological status, 17.2% presented markers of exposure (HBsAg and/or anti‐HBc+), 35.6% were susceptible, and 47.1% had probable vaccine‐induced immunity (isolated anti‐HBs+). Male sex was associated with HBV exposure, whereas female sex was associated with susceptibility (*p* < 0.001 and *p* = 0.036, respectively). Younger age (≤ 50 years) was related to vaccination status (*p* = 0.027). A history of jaundice was associated with HBV exposure (*p* = 0.030) and lower vaccination rates (*p* = 0.034), and psychotropic substance use with incomplete vaccination status (*p* = 0.014). Complete vaccination coverage (anti‐HBs+ and/or ≥ 3 doses on the vaccination card) was low (51.2%), particularly among drug users. No anti‐HDV reactivity was detected among HBsAg‐positive individuals.

**Conclusion:**

HBV and HCV prevalence was higher than the national average in hemodialysis, with gender disparities between exposed and susceptible and low vaccination coverage, especially in drug users. Although no anti‐HDV reactivity was found, investigating hepatitis D in this population remains relevant to improving understanding of its geographical distribution and clinical impact. It is recommended to reinforce screening, vaccination, and safety protocols in hemodialysis clinics.

## 1. Introduction

In Brazil, chronic viral hepatitis B and C remain a major public health concern. According to the 2025 epidemiological report published by the Brazilian Ministry of Health, 302,351 cases of hepatitis B were reported between 2000 and 2024, while 342,328 and 4722 cases of hepatitis C and hepatitis D, respectively, were recorded during the same period [1].

Chronic kidney disease (CKD) represents a growing global public health burden. Current data from the International Society of Nephrology estimate that more than 850 million people live with CKD globally, many of whom reside in low‐ and middle‐income countries, where social development directly impacts diagnosis, access to treatment, and quality of care [2, 3]. In Brazil, between 2019 and 2023, 305,632 men and 275,170 women received care for CKD in Primary Healthcare, reflecting the increasing healthcare burden associated with this condition rather than solely an increase in its diagnosis, highlighting the substantial burden of CKD on public health [4].

Hemodialysis patients are considered a high‐risk group for HBV and HCV infection due to repeated vascular access, frequent healthcare exposure, blood transfusions, and the potential for nosocomial transmission during dialysis procedures [5]. In addition, CKD patients may exhibit reduced antibody responses to hepatitis B vaccination due to immune dysfunction associated with uremia, chronic inflammation, and impaired cellular and humoral immunity, increasing their susceptibility to HBV infection [6]. Moreover, chronic viral hepatitis has been associated with kidney dysfunction. A North American study involving more than 70,000 patients with hepatitis B demonstrated an association between HBV infection and an increased risk of CKD [7]. This association is biologically plausible, as HBV‐associated kidney injury is primarily mediated by the deposition of circulating immune complexes containing viral antigens within the glomeruli, resulting in complement activation, immune‐mediated glomerular injury, and the development of HBV‐associated glomerular diseases. Additional mechanisms, including direct viral cytopathic effects, chronic inflammation, and immune dysregulation, may also contribute to renal injury [7, 8]. A similar relationship has been described for hepatitis C, in which HCV infection may act both as a cause and a consequence of CKD. Among patients with HCV‐associated mixed cryoglobulinemia, renal manifestations occur in approximately 20%–35% of cases, with membranoproliferative glomerulonephritis representing the most common HCV‐associated glomerular disease [9].

According to the multinational Dialysis Outcomes and Practice Patterns Study (DOPPS), which included hemodialysis centers in France, Germany, Italy, Japan, Spain, the United Kingdom, and the United States, the mean prevalence of HCV infection was 13.5%, ranging from 2.6% to 22.9% across participating countries [9, 10]. In Brazil, 2017 data from the Brazilian Society of Nephrology reported a prevalence of 0.8% for hepatitis B and 3.3% for hepatitis C in national hemodialysis clinics [11]. In southeastern Brazil, a study conducted in Rio de Janeiro identified 5.9% HBsAg reactivity among hemodialysis patients [12].

Epidemiological data on hepatitis D among individuals with CKD remain scarce. In Brazil, only one study has investigated HDV infection in hemodialysis patients and renal transplant recipients. Conducted in São Paulo, the study reported a 1.7% prevalence of anti‐HDV among HBsAg‐positive individuals [13].

The lack of updated studies on the seroprevalence of chronic viral hepatitis B, C and D among patients with CKD in Brazil, particularly in specific macroregions, such as the Northeast, underscores the need and relevance of this investigation. The Northeast region presents distinct sociodemographic and healthcare access characteristics, as well as historically different patterns of viral hepatitis distribution compared to other Brazilian regions, reinforcing the importance of region‐specific epidemiological data. This study focuses on the evaluation of serological markers for hepatitis B (HBsAg and anti‐HBc), the assessment of hepatitis B vaccination coverage (anti‐HBs), the detection of hepatitis C infection through total anti‐HCV, and hepatitis D through total anti‐HDV in this high‐risk population. Furthermore, sociodemographic, clinical, and behavioral factors will be analyzed in relation to distinct hepatitis B serological profiles.

## 2. Materials and Methods

### 2.1. Study Design, Ethical Aspects, and Aim

This observational cross‐sectional study was approved by the Ethics Committee of the Oswaldo Cruz Foundation (approval number 11177119.8.0000.5248) and conducted in accordance with the principles of the 1964 Declaration of Helsinki and its subsequent amendments. Written informed consent was obtained from all participants prior to enrollment. The study was based on data collected in June 2022 from patients with CKD undergoing hemodialysis at two dialysis clinics in Fortaleza, Ceará, Northeast Brazil. The aim of this work was to determine the seroprevalence of hepatitis B, C, and D viruses (HBV, HCV, and HDV) and to investigate potential associations between hepatitis B serological markers and sociodemographic, clinical, and behavioral risk factors among chronic hemodialysis patients in Northeast Brazil.

### 2.2. Study Population and Biological Material Collection

The study population consisted of 293 individuals aged 18 years or older, and no participants were excluded after data collection. The inclusion criteria were age ≥ 18 years, diagnosis of CKD, and patients undergoing hemodialysis. The exclusion criteria were refusal to provide informed consent, history of renal transplantation, current use of immunosuppressive therapy, or inadequate biological samples for laboratory analysis. Blood samples were collected from all participants by peripheral venipuncture under aseptic conditions.

The sample was selected by convenience and consisted of participants who met the eligibility criteria and agreed to participate during the data collection period. Therefore, this was a nonprobability sample, which does not allow inferences to the target population of patients undergoing hemodialysis at the two clinics included in the study.

### 2.3. Serological Tests for Viral Hepatitis

The interpretation of serological and molecular results (HBV and HCV viral loads) for the hepatitis B, C and D diagnoses was based on protocols established by the European Association for the Study of the Liver (EASL) [14–16]. Diagnostic tests for hepatitis B, C and D were performed to characterize the study population using a commercial chemiluminescence assay. The tests were performed on the LIAISON XL device (DiaSorin Saluggia, Italy) for HBsAg, total anti‐HBc, anti‐HBs, anti‐HCV, and anti‐HDV. For HBsAg, results considered reactive have relative light units (RLU) above 0.30 international units per milliliter (IU/mL). Samples that tested positive for HBsAg were subjected to total anti‐HDV with an RLU above 1.0 for detection. As for anti‐HBs, RLU was equal to or higher than 10 IU/mL. For anti‐HBc and anti‐HCV, the detection limit (in RLU) is 1.0 for both assays. Anti‐HBc is a competitive assay, where reactive results are defined as values below 1.0, while anti‐HCV reactivity is indicated by values above 1.0. All tests have sensitivity and specificity in the range of 99.5%–100%.

### 2.4. Molecular Tests for Viral Hepatitis

All reactive samples for HBsAg or anti‐HCV were submitted to viral load quantification through real‐time PCR by Cobas 6800/8800 (Roche, Switzerland). The HBV polymerase (pol) gene and the HCV 5′ untranslated (UTR) region comprehend the amplified targets. The methodology is based on standard curves established with different controlled viral loads. Based on the measurement of the emitted fluorescence and the standard curve, the absolute concentration of the viral load is calculated in IU/mL. The quantification ranges are 10 to 1,000,000,000 IU/mL for HBV and 15 to 100,000,000 IU/mL, converted to log 10 for HCV.

### 2.5. Variables

The following variables were analyzed: sex at birth (male and female), age group defined using the median value as the cutoff (≤ 50 years and > 50 years), educational level (elementary school, high school, and higher education), and household income categorized according to the equivalent value in U.S. dollars using the 2025 exchange rate (up to two minimum wages and more than two minimum wages). Potential epidemiological risk factors, healthcare‐related exposures, comorbidities, and clinical characteristics associated with hepatitis B were assessed, including self‐reported history of jaundice (yellowing of the skin or eyes), blood transfusion, previous surgery, hospitalization within the previous 3 months, substance use, alcohol consumption, high blood pressure, cardiovascular disease, diabetes mellitus, medication use, and symptoms suggestive of liver disease. Jaundice and symptoms suggestive of liver disease were included as indicators of previous or current hepatic disease. High blood pressure, cardiovascular disease, and diabetes mellitus were considered comorbidities, whereas medication use was evaluated as a proxy for healthcare utilization and potential exposure to invasive medical procedures. All information was collected through a structured questionnaire using binary (yes/no) response options.

Participants were classified into four categories based on the hepatitis B test results: (1) exposure to HBV, defined as positivity for markers of exposure or infection (HBsAg and/or anti‐HBc); (2) susceptible, a group comprised of individuals who tested negative for HBsAg, anti‐HBc, and anti‐HBs; (3) vaccinated individuals, defined as isolated positivity for the anti‐HBs marker; and (4) vaccination card and immune response completed, individuals with positive anti‐HBs serological markers and/or who reported receiving three or more doses of the vaccine in the vaccination card.

### 2.6. Statistical Analysis

Absolute and relative frequencies, together with their 95% confidence intervals (95% CI), were calculated and compared according to the distribution of variables by exposure, susceptibility, vaccination status, and hepatitis B diagnosis. Categorical variables were compared between groups using the chi‐square test or Fisher’s exact test. A *p* value < 0.05 was considered statistically significant. Statistical analyses were performed using R software Version 4.5.0 (R Foundation for Statistical Computing, Vienna, Austria).

## 3. Results

A total of 293 participants were included in the study. Sociodemographic characteristics and potential risk factors associated with liver disease are presented in Table 1. Most of the study population was over 50 years of age, comprising 194 individuals (66.2%). Most participants were male (63.1%, 185/293), had an educational level up to elementary school (50.5%, 139/293), and reported a monthly income below USD 538.67 (76.1%, 194/293). The most common epidemiological variables related to hepatitis risk were a history of medication use (87.7%, 257/293), followed by high blood pressure (74.0%, 216/293) and a history of surgery (68.8%, 201/293).

**TABLE 1:** Sociodemographic characteristics and vulnerability factors for viral hepatitis among patients undergoing hemodialysis.

Variable	*N* (%)	CI 95%
	*n* = 293	
*Age*		
Up to 50 years old	99 (33.8)	28.6–39.4
Over 50 years old	194 (66.2)	60.6–71.4

*Sex*		
Female	108 (36.9)	31.5–42.5
Male	185 (63.1)	57.5–68.5

*Education*		
Up to elementary school	139 (50.5)	41.8–53.2
High school	99 (36.0)	28.6–39.4
Higher education	37 (13.5)	9.3–16.9

*Monthly income*		
≤ 538.67 USD	194 (76.1)	60.6–71.4
≥ 538.67 USD	61 (23.9)	16.6–25.8

*Epidemiological variables related to hepatitis risk*		
Yellow skin or eyes	39 (13.3)	9.9–17.7
Blood transfusion	109 (37.2)	31.9–42.9
Previous surgery	201 (68.6)	63.1–73.6
High blood pressure	216 (73.7)	68.4–78.4
Regular use of prescription medications	257 (87.7)	83.5–91.0
Cardiovascular	66 (22.5)	18.1–27.6
Diabetes mellitus	61 (20.8)	16.6–25.8
Symptom of liver disease	30 (10.2)	7.3–14.2
Hospitalization in the past 3 months	30 (10.2)	7.3–14.2
Substance use	12 (4.1)	2.4–7.0
Alcohol consumption	36 (12.3)	9.0–16.5

*Note:* The *n* presented refers to the amount of information available in each analysis. Percentages are based on available data for each variable.

The distribution of reactive serological markers for hepatitis B, C and D is presented in Table 2. For hepatitis B, 7 out of 293 individuals (2.4%) were reactive for HBsAg, 46.4% (136/293) were reactive only for anti‐HBs, and 13.0% (38/293) had both anti‐HBc and anti‐HBs reactivity. Additionally, 35.2% (103/293) presented no reactivity for any hepatitis B markers. Of the individuals who tested reactive for HBsAg, none tested positive for anti‐HDV. Regarding hepatitis C, 4.1% (12/293) were reactive for anti‐HCV. Regarding the mean viral load, HCV RNA levels averaged 28 (±27.98) IU/mL, while the mean HBV DNA load was 8.84 (±12.10) IU/mL.

**TABLE 2:** Serological profile of hepatitis B, C, and D and mean viral load of hepatitis B and C molecular markers in the study population.

Markers	*n* (%)	CI 95%
HBsAg	7 (2.4%)	1.2–4.8
Anti‐HDV	0 (0%)	—
Anti‐HBs+/anti‐HBc and HBsAg‐	136 (46.4%)	40.8–52.1
Anti‐HBc and anti‐HBs+	38 (13%)	9.6–17.3
HBsAg, anti‐HBc, and anti‐HBs‐	103 (35.2%)	29.9–40.8
Anti‐HCV	12 (4.1%)	2.4–7
Mean DNA of HBV (±SD)	8.84 IU/mL (±12.10)	0–20.3
Mean RNA of HCV (±SD)	28 IU/mL (±27.98)	10.2–45.8

Abbreviation: SD, standard deviation.

The association between the participant categories and the sociodemographic variables and potential risk factors for hepatitis B is presented in Table 3. For the vaccination category, a statistically significant association was observed between age and hepatitis B vaccination status, with a higher number of vaccinated individuals in the age group up to 50 years (39.7%, 54/136) compared to unvaccinated individuals in the same age group (27.5%, 42/153). A higher proportion of men were exposed to HBV (86.0%, 43/50). The prevalence of susceptibility was higher among women (44.7%, 46/103) compared to the opposite profiles (*p* > 0.05). Regarding hepatitis risk‐related variables, the percentage with a history of jaundice was statistically significant in 22.0% (11/50) of those who were exposed when compared to those who were not exposed (11.3%, 27/240). Regarding vaccination status, a statistically significant association was observed (*p* = 0.034). The prevalence was 17.0% (26/153) among unvaccinated individuals, compared with 8.8% (12/136) among vaccinated individuals. As for the relationships that had *p* values close to 0.05 between the variables, a higher percentage of individuals with until elementary school education was observed among those who were exposed (48%, 24/50) in comparison to susceptible individuals (50.5%, 52/103). About nonsusceptible individuals, they presented a higher percentage of hepatic symptoms (11.8%, 22/186).

**TABLE 3:** Sociodemographic characteristics and vulnerabilities among individuals undergoing dialysis, according to exposure status, vaccination, and susceptibility for hepatitis B.

Variables	Exposure (HBsAg and/or anti‐HBc+)			Susceptibility (HBsAg, anti‐HBc, and anti‐HBs‐)			Vaccination (only anti‐HBs +)		
	*N* (%)	CI 95%	*p*_value	*N* (%)	CI 95%	*p*_value	*N* (%)	CI 95%	*p*_value
	50 (17.2)	13.3–22.0		103 (35.6)	30.3–41.3		136 (47.1)	41.4–52.8	
*Age*									
Up to 50 years old	13 (26.0)	15.9–39.6	0.219	29 (28.2)	20.4–37.5	0.173	54 (39.7)	31.9–48.1	**0.027**
Over 50 years old	37 (74.0)	60.4–84.1		74 (71.8)	62.5–79.6		82 (60.3)	51.9–68.1	

*Gender*									
Female	7 (14.0)	7.0–26.2	**0.000**	46 (44.7)	35.4–54.3	**0.036**	53 (39.0)	31.2–47.4	0.445
Male	43 (86.0)	73.8–93.0		57 (55.3)	45.7–64.6		83 (61.0)	52.6–68.8	

*Education*									
Up to elementary school	24 (54.5)	40.1–68.3	0.308	52 (53.1)	43.3–62.6	0.760	61 (47.3)	38.9–55.9	0.197
High school	12 (27.3)	16.3–41.8		33 (33.7)	25.1–43.5		54 (41.9)	33.7–50.5	
Higher education	8 (18.2)	9.5–32.0		13 (13.3)	7.9–21.4		14 (10.9)	6.6–17.4	

*Monthly income*									
≤ 538.67 USD	30 (69.8)	54.9–81.4	0.277	65 (74.7)	64.7–82.7	0.627	97 (80.2)	72.2–86.3	0.185
≥ 538.67 USD	13 (30.2)	18.6–45.1		22 (25.3)	17.3–35.3		24 (19.8)	13.7–27.8	

*Epidemiological variables related to hepatitis risk*									
Yellow skin or eyes	11 (22.0)	13.3–36.5	**0.030**	15 (14.7)	9.1–22.9	0.598	12 (8.8)	5.1–14.8	**0.034**
Blood transfusion	22 (44.0)	31.9–58.7	0.226	38 (37.3)	28.5–46.9	0.960	46 (34.1)	26.6–42.4	0.322
Previous surgery	31 (63.3)	49.3–75.3	0.353	73 (70.9)	61.5–78.8	0.561	94 (69.1)	60.9–76.3	0.898
High blood pressure	38 (77.6)	64.1–87.0	0.539	81 (78.6)	69.8–85.5	0.176	94 (69.1)	60.9–76.3	0.076
Regular use of prescription medications	41 (82.0)	69.2–90.2	0.187	95 (92.2)	85.4%–96%	0.072	117 (86.0)	79.2–90.9	0.462
Cardiovascular	8 (16.0)	8.3–28.5	0.231	28 (27.2)	19.5–36.5	0.155	29 (21.3)	15.3–28.9	0.653
Diabetes mellitus	9 (18.0)	9.8–30.8	0.605	22 (21.4)	14.5–30.2	0.852	29 (21.3)	15.3–28.9	0.824
Symptom of liver disease	8 (16.7)	8.7–29.6	0.156	6 (6.2)	2.9–13.0	0.085	14 (11.6)	7.0–18.5	0.625
Hospitalization in the past 3 months	7 (14.0)	7.4–27.7	0.301	7 (7.0)	4.0–13.7	0.131	15 (12.1)	7.5–19.0	0.494
Substance use	1 (2.1)	0.4–11.1	0.697	4 (4.0)	6.0–9.7	1.000	6 (4.5)	2.1–9.5	0.624
Alcohol consumption	7 (14.3)	7.1–26.7	0.623	10 (9.8)	5.4–17.1	0.349	18 (13.3)	8.6–20.1	0.593

*Note:* Values in bold are associations that presented statistical significance (*p* < 0.05). The *n* presented refers to the amount of information available in each analysis. Percentages are based on available data for each variable.

An analysis was conducted to assess the association between sociodemographic and hepatitis risk‐related variables and complete vaccination status, as verified by the vaccination cards, and their relationship with serological markers of vaccine‐induced immunity (Table 4). Statistical significance was observed between a history of psychotropic substances and an incomplete vaccination history, with a higher percentage of users who do not have complete vaccination protection (7.0%, 10/143) in comparison with 1.3% (2/150) who had complete immunization according to their vaccination card. For *p* values that approached 0.05, history of hospitalization in the last 3 months of the data collection period and diabetes were more prevalent among the unimmunized, with 25.2% (36/143) and 13.3% (19/143), respectively, compared to the immunized.

**TABLE 4:** Distribution of hepatitis B vaccination (serological and/or declared) by sociodemographic and risk characteristics.

Variables	Vaccination card and immune response completed				Test
	No		Yes		
	*n* (%)	CI 95%	*n* (%)	CI 95%	
	143 (48.8)	43.1–54.5	150 (51.2)	45.5–56.9	
*Age*					
Over 50 years old	97 (67.8)	59.8–74.9	97 (64.7)	56.7–71.9	0.5669
Up to 50 years old	46 (32.2)	25.1–40.2	53 (35.3)	28.1–43.3	

*Gender*					
Female	48 (33.6)	26.3–41.6	60 (40.0)	32.5–48.0	0.2539
Male	95 (66.4)	58.4–73.7	90 (60.0)	52.0–67.5	

*Education*					
Up to elementary school	71 (51.4)	43.2–59.6	68 (49.6)	41.4–57.9	0.9521
High school	49 (35.5)	28.0–43.8	50 (36.5)	28.9–44.8	
Higher education	18 (13.0)	8.4–19.7	19 (13.9)	9.1–20.6	

*Monthly income*					
≤ 538.67 USD	100 (78.1)	70.2–84.4	94 (74.0)	65.8–80.9	0.4418
≥ 538.67 USD	28 (21.9)	15.6–29.8	33 (26.0)	19.1–34.2	

*Epidemiological variables related to hepatitis risk*					
Yellow skin or eyes	23 (16.2)	11.0–23.1	16 (10.8)	6.8–16.8	0.1789
Blood transfusion	47 (33.3)	26.1–41.5	62 (41.6)	34.0–49.6	0.1458
Previous surgery	94 (66.2)	58.1–73.5	107 (71.3)	63.6–78.0	0.3436
High blood pressure	107 (75.4)	67.7–81.7	109 (72.7)	65.0%79.2	0.6012
Regular use of prescription medications	128 (89.5)	83.4–93.5	129 (86.0)	79.5–90.7	0.3602
Cardiovascular	33 (23.1)	16.9–30.6	33 (22.0)	16.1–29.3	0.8254
Diabetes mellitus	36 (25.2)	18.8–32.9	25 (16.7)	11.6–23.4	0.0730
Symptom of liver disease	15 (11.6)	7.2–18.3	15 (10.7)	6.6–16.9	0.8120
Hospitalization in the past 3 months	19 (14.5)	9.5–21.5	11 (7.6)	4.3–13.2	0.0682
Use of psychotropic substances	10 (7.2)	4.0–12.7	2 (1.4)	0.4–4.9	**0.0144**
Alcohol consumption	17 (12.1)	7.7–18.6	19 (12.7)	8.3–18.9	0.8925

*Note:* Values in bold are associations that presented statistical significance (*p* < 0.05). The *n* presented refers to the amount of information available in each analysis. Percentages are based on available data for each variable.

## 4. Discussion

In this study, a high seroprevalence of hepatitis B (HBsAg positive: 2.4%) and hepatitis C (anti‐HCV reactive: 4.1%) was found among patients treated at hemodialysis clinics in Fortaleza, Ceará, as compared to the national prevalence for hepatitis B (0.8%) and hepatitis C (2.2%) in a study using the database of the Brazilian Society of Nephrology [11]. Although the latter was a nationwide study, this prevalence is noteworthy due to the divergence in values between markers measured individually in units in specific regions of Brazil. Supporting this observation, a review analyzed the prevalence of hepatitis C among hemodialysis patients described in studies covering a 20‐year period (between 1989 and 2019) in Brazil [18]. This analysis suggested an increase in anti‐HCV reactivity from 2010 among patients with kidney disease, in contrast to the decline observed between 1989 and 2001. This review also highlighted a heterogeneous distribution of HCV infection across Brazil, with the Northeast Region demonstrating the lowest anti‐HCV reactivity rate (7.9%) in studies conducted after 2001, compared to other macroregions of Brazil [17]. Therefore, the 4% reactivity rate for anti‐HCV found in Fortaleza is notable, as it exceeds the national average and is close to the detection rate in current studies on the topic in the Northeast Region.

When evaluating hemodialysis centers in another state in Northeast Brazil (Pernambuco), a seroprevalence similar to ours was observed, with 3.3% HBsAg positivity among patients [18]. As for the state of Paraíba, a study in hemodialysis clinics described a seroprevalence of 0.2% for HBV and 2% for HCV in the state capital, João Pessoa [19]. In the present study, among patients with HBsAg reactivity, no anti‐HDV positivity was identified. In fact, in a national overview of hepatitis D prevalence in Brazil, the Northeast Region ranks second to last, with 0.8% of positive cases, whereas no cases were identified in the Southern Region within the analyzed sample [20]. Despite the low prevalence observed in our study, investigating HDV infection in patients undergoing hemodialysis remains clinically relevant. HDV is an obligate satellite virus of HBV and is associated with more severe forms of viral hepatitis, accelerated progression to cirrhosis, and an increased risk of hepatocellular carcinoma compared with HBV monoinfection. In addition, patients receiving hemodialysis are considered a high‐risk population for blood‐borne viral infections because of repeated vascular access and frequent healthcare exposure. However, epidemiological data on HDV in this population remain scarce, particularly outside endemic regions, reinforcing the need for additional studies to better characterize the distribution of HDV infection among patients with CKD [13, 21]. A Brazilian cross‐sectional study evaluating HDV infection among 117 HBsAg‐positive patients undergoing hemodialysis or renal transplantation in São Paulo, southeastern Brazil, detected anti‐HDV antibodies in only two individuals (1.7%), and no cases of active HDV infection were identified by HDV RNA testing [13].

These data reinforce the need for hemodialysis clinics to screen patients with viral hepatitis B, C and D to prevent intra‐center contamination. The measures include patient testing, completion of hepatitis B vaccination, allocation of dedicated rooms for procedures involving patients with viral hepatitis, and proper decontamination of equipment and materials used during treatment. Furthermore, it is important to consider that patients undergoing hemodialysis may have a reduced immune response to hepatitis B vaccination and may not produce adequate levels of protective anti‐HBs antibodies, which further increases their vulnerability to HBV infection and reinforces the need for postvaccination serological monitoring [6, 12, 22]. Achieving the World Health Organization’s goal of eliminating hepatitis B and C by 2030 remains a major challenge in Brazil, particularly among high‐risk populations such as patients undergoing hemodialysis, reinforcing the need for targeted microelimination strategies in this population [17]. The low viral loads observed in the study are consistent with patterns seen in treated or immune‐controlled chronic hepatitis, though treatment status was not confirmed in this cohort [23, 24].

The bivariate analysis revealed significant differences in exposure and susceptibility to hepatitis B among patients with CKD. A higher proportion of exposed individuals were male, while among those susceptible, the majority are women. Although the absolute number of susceptible men was higher, women were proportionally overrepresented in this group. A Brazilian study assessing the serological profile of hepatitis B markers in hemodialysis patients from Southeast Brazil reported a higher prevalence of HBsAg among men (6.1%; 21/346) compared to 17 women (5.7%; 17/298). In the same study, anti‐HBc was also more frequently detected in men (3.5%, 12/346) [12].

Our data reinforce the need for increased attention to hepatitis B among men, given the higher number of positive cases in this group. This finding is associated with lower rates of medical consultations and reduced acceptance of testing for viral hepatitis, which hinders early diagnosis and treatment in this population [25, 26]. Furthermore, recent data from the Brazilian Dialysis Survey (BDS) indicated that most dialysis patients in Brazil are male (59%), which may have contributed to the results observed in this study [27]. Epidemiological bulletins from the Brazilian Ministry of Health reported that most hepatitis B cases occur among men (55%). At the same time, this gender tends to be less inclined to seek care proactively at primary healthcare centers, often seeking assistance only when symptoms become more severe [25, 28]. The absence of hepatitis B markers among women reinforces the need for vaccination among them in vulnerable groups. A study that evaluated women in vulnerable situations found that 52.2% remained susceptible (neither vaccinated nor infected) [29]. Despite this, when hemodialysis patients were evaluated, our data differed from the literature. Villar and collaborators (2022) suggested that most women on hemodialysis had higher anti‐HBs reactive concentrations [12].

Our study highlights a concentration of individuals who reported use of psychotropic substances and who are not fully immunized against hepatitis B, either by vaccination card or by anti‐HBs reactivity (*p* < 0.05). This finding suggests a failure in mandatory immunization for hepatitis B among hemodialysis patients, which might increase the risk of aggravation, especially considering individuals who had a history of injectable or inhaled drug use. A study that evaluated vaccination coverage among drug users in Rio de Janeiro found that 65% of the study population was not covered [30]. The percentage of complete hepatitis B immunization among hemodialysis patients in our study was low (51.2%). This finding is inconsistent with the policies of the Brazilian National Immunization Program, which have mandated hepatitis B vaccination for hemodialysis patients since 2004 [31]. To date, there are no known studies evaluating illegal drug users undergoing hemodialysis in association with low vaccination coverage, underscoring the originality of our study and its emphasis on complete vaccination for these doubly vulnerable individuals. The proximity to the threshold of statistical significance indicates that the highest number of hospital admissions in the past 3 months also occurred among individuals who were not fully immunized. This may reflect a history of liver damage among those who may have hepatitis B and required hospitalization [32].

Also, there was statistical significance for the presence of jaundice among those exposed and vaccination (*p* = 0.030 and 0.034, respectively), suggesting that a history of jaundice may be related to previous exposure to hepatitis B and probably absence of previous vaccination. It is well known that jaundice is one of the main symptoms in the acute phase of HBV infection, while in the chronic phase, this symptom is associated with advanced stages of liver damage [33]. Interestingly, individuals with jaundice had a lower prevalence of hepatitis B vaccination, which increases attention to the vulnerability of these individuals. Unvaccinated people are more exposed and may have already acquired HBV, presenting symptoms of abnormal liver function [23].

Among individuals exposed to HBV, there was a higher percentage of people with low levels of education (24/44%–54.5%), corroborating a previous study by Marriz et al., 2024, which also evaluated sociodemographic data among individuals with hepatitis B in a population from the state neighboring the population of our study, the Brazilian state of Ceará, where 85% (6/7) of these individuals had completed high school or less [34]. Another study observed high exposure to hepatitis B among individuals living in extreme poverty in central–western Brazil [35]. Low education is associated with the socioeconomic status of individuals, which demonstrates an increased need for attention and health education among these individuals [34]. The presence of liver disease symptoms among nonsusceptible individuals may reflect sequelae of prior hepatitis B infection, particularly in those with serological markers indicating previous exposure or chronic infection [32]. The sociodemographic profile observed in the study population, predominantly men, with a mean age above 50 years, low education attainment and income, and a high prevalence of hypertension, medication use, and prior surgeries, reflects a common pattern among patients undergoing hemodialysis in public centers in Brazil, consistent with findings from other studies on Brazilian hemodialysis patients [11, 12, 19]. Although it provides valuable information on the seroprevalence of hepatitis B and C in hemodialysis patients, this study has limitations, such as convenience sampling without a prior sample size calculation, the possibility of expanding the investigation to a larger number of clinics in the region, and the absence of molecular genetic sequencing tests to investigate the viral genotype profile and possible transmission routes. Additionally, antiviral treatment status was not confirmed among HBV‐ and HCV‐positive individuals, which may have influenced the interpretation of viral load findings. Furthermore, the study may be subject to information and recall bias, as some data were obtained through questionnaires and vaccination records that may have been incomplete or inaccurately reported by participants.

Another limitation of this study is its cross‐sectional design, which precludes establishing a temporal relationship between hemodialysis treatment and blood‐borne viral infections. Therefore, it is not possible to determine whether HBV or HCV infections were acquired before the initiation of hemodialysis or during treatment. HCV RNA testing was not performed in anti‐HCV–negative participants; consequently, cases of seronegative or occult HCV infection may have been missed, potentially leading to an underestimation of the true prevalence of HCV infection in this population. Nevertheless, anti‐HCV serology remains the primary screening method recommended and widely used for the epidemiological assessment of HCV infection, supporting the validity and relevance of the present findings. Longitudinal studies incorporating universal HCV RNA testing are needed to better clarify the timing of infection and improve the detection of active HCV infection among patients undergoing hemodialysis.

## 5. Conclusion

This study demonstrated a high prevalence of hepatitis B and hepatitis C among patients undergoing hemodialysis in Northeast Brazil, with rates exceeding the national averages, providing updated epidemiological evidence for a region where data remain limited. The findings revealed distinct patterns of HBV exposure and susceptibility according to sex, as well as suboptimal hepatitis B vaccination coverage, particularly among drug users, highlighting persistent gaps in the implementation of recommended preventive measures for this high‐risk population. Hepatitis delta was not detected; however, continued surveillance remains important to better define its epidemiology in vulnerable groups. Overall, our findings provide updated epidemiological evidence on viral hepatitis among patients undergoing hemodialysis in Northeast Brazil and identify important gaps in hepatitis B vaccination coverage. Strengthening vaccination, expanding surveillance, and implementing targeted microelimination strategies in this high‐risk population are essential to support Brazil’s progress toward the World Health Organization goal of eliminating hepatitis B and C as public health threats by 2030.

## Funding

Coordination for the Improvement of Higher Education Personnel (CAPES), Oswaldo Cruz Foundation, Oswaldo Cruz Institute, and Carlos Chagas Filho Foundation for Research Support of the State of Rio de Janeiro (FAPERJ).

## Conflicts of Interest

The authors declare no conflicts of interest.

## Data Availability

The data supporting the conclusions of this study are available upon reasonable request to the corresponding author.
